# Opposite Effects of SiO_2_ Nanoparticles on the Local *α* and Larger-Scale *α*’ Segmental Relaxation Dynamics of PMMA Nanocomposites

**DOI:** 10.3390/polym11060979

**Published:** 2019-06-03

**Authors:** Na Wang, Xuebang Wu, C.S. Liu

**Affiliations:** 1 Key Laboratory of Materials Physics, Institute of Solid State Physics, Chinese Academy of Sciences, Hefei 230031, China; nwang@mail.ustc.edu.cn; 2 Department of Materials Science and Engineering, University of Science and Technology of China, Hefei 230026, China

**Keywords:** polymer nanocomposite, relaxation dynamics, mechanical spectroscopy, local heterogeneity

## Abstract

The segmental relaxation dynamics of poly(methyl methacrylate)/silica (PMMA/SiO_2_) nanocomposites with different compositions ($\phi_{{\mathrm{SiO}}_{2}}$) near and above the glass transition temperature were investigated by mechanical spectroscopy. At $\phi_{{\mathrm{SiO}}_{2}}$ ≤ 0.5%, the *α* peak temperature hardly changes with $\phi_{{\mathrm{SiO}}_{2}}$, but that of *α*’ relaxation composed of Rouse and sub-Rouse modes decreases by 15 °C due to the increase of free volume. At $\phi_{{\mathrm{SiO}}_{2}}$ ≥ 0.7%, both *α* and *α*’ relaxations shift to high temperatures because of the steric hindrance introduced by nanoparticle agglomeration. On the other hand, with increasing $\phi_{{\mathrm{SiO}}_{2}}$, the peak height for *α* relaxation increases at $\phi_{{\mathrm{SiO}}_{2}}$ ≤ 0.5% and then decreases at $\phi_{{\mathrm{SiO}}_{2}}$ ≥ 0.7%, but that for *α*’ relaxation shows an opposite behavior. This is because at low $\phi_{{\mathrm{SiO}}_{2}}$, the short-chain segments related to *α* relaxation can easily bypass the particles, but the longer-chain segments related to *α*’ relaxation cannot. At high $\phi_{{\mathrm{SiO}}_{2}}$, the polymer chains were bound to the nanoparticles due to the physical adsorption effect, leading to the decrease of relaxation unit concentration involved in *α* relaxation. However, the dissociation of those bonds with heating and the concentration heterogeneity of polymer chains result in the increase of peak height for *α*’ relaxation.

## 1. Introduction

Compared with the traditional polymer composites, polymer nanocomposites produced by adding various nanoparticles to polymer matrices have received much attention due to their significantly improved physicochemical properties, such as mechanical performance or electrical conductivity [1,2,3]. To date, a number of studies on modifications of nanoparticles such as size, fraction, and surface decoration have been performed to reveal the effect of nanoparticles and uncover the mechanisms of the properties’ enhancement of polymer nanocomposites. It has been shown that the interfacial polymer layer surrounding nanoparticles plays an important role in changing the dynamics and macroscopic properties of polymer nanocomposites [4,5]. The surface area of nanoparticles increases with the decreasing particle size, which leads to the increase of interfacial layer volume fraction and ultimately slows down the relaxation process [6]. However, recent studies reported that nanoparticles usually play a plasticizer role in the polymer matrix if the particles are small enough to result in reversing the dynamics [7,8]. Meanwhile, the interaction between nanoparticles and polymer chains has a great influence on the segmental mobility and dynamics of polymer nanocomposites. If the interaction is attractive, the glass transition process of polymer nanocomposites becomes broader and the glass transition temperature (*T_g_*) shifts to higher temperature, which indicates a slower relaxation process due to the immobilization and restriction of polymer segments by nanoparticles [9,10]. In addition, some polymer nanocomposites such as poly(methyl methacrylate) (PMMA)/organically modified MgAl nanocomposites show two *T_g_* values, and the higher *T_g_* corresponds to the dynamics of polymer chains at the interfacial layer [11,12]. On the other hand, if the interaction is repulsive, the addition of nanoparticles will increase the free volume at low fractions, resulting in the decrease of *T_g_* and the acceleration of the segmental relaxation process [13]. However, the addition of such nanoparticles also introduces steric hindrance, so the segmental motion of the composites will be slowed down at high nanoparticle content [14,15]. Bogoslovov et al. reported no change in segmental relaxation dynamics associated with *T_g_* for polymer nanocomposites [16,17]. Therefore, the interaction between nanoparticles and polymers can be adjusted to regulate the desired macroscopic properties of polymer nanocomposites [18]. Understanding the segmental dynamics of polymer nanocomposites is of critical importance since it is essential to optimize and obtain the desired performance of materials. However, the effect of nanoparticles on segmental and chain relaxation dynamics is far from being understood, especially the dynamics of longer-chain segments such as sub-Rouse modes.

Besides the glass transition, in amorphous polymers, there exists another type of larger-scale segmental relaxation dynamics, namely *α*’ relaxation, composed of sub-Rouse modes and Rouse modes, which is related to the main transition from glass-to-rubber softening dispersion [19,20]. The sub-Rouse modes have been investigated by many experimental techniques, such as mechanical spectroscopy, photo correlation spectroscopy, and dielectric relaxation spectroscopy [21,22,23]. The sub-Rouse modes involve numbers of repeat units on a chain length-scale intermediate between the *α* relaxation and the Rouse modes. It was found that the property of the sub-Rouse modes resembles that of the *α* relaxation, showing a similar cooperative nature to the *α*-process, albeit to a lesser degree [24]. What’s more, the longer chains’ motion is associated with material flow and processing performance. Therefore, understanding the nature of longer-chain segmental dynamics in polymer nanocomposites not only enriches the current understanding of the glass–rubber transition, but also has practical significance for processing technology.

The poly(methyl methacrylate)/silica (PMMA/SiO_2_) polymer nanocomposites with excellent mechanical properties [25,26] and thermal stability [27] have attracted extensive attention, and their segmental dynamics have been widely investigated by various techniques [28,29,30]. The previous studies show that the addition of silica nanoparticles leads to the broadening of the glass transition process, the increase of *T_g_*, and the slowing down of the *α* relaxation process due to the attractive interaction between silica nanoparticles and PMMA [31,32,33]. Song et al. reported that the rigid amorphous fraction layer around silica nanoparticles leads to adjacent molecular packing frustration, resulting in the significant reduction of *T_g_* and the segmental acceleration of the PMMA matrix [34]. However, there are still few studies on the longer-chain segmental relaxation dynamics of PMMA/SiO_2_ nanocomposites in the whole glass-to-rubber softening region. In this work, the local and longer-chain segmental dynamics of PMMA/SiO_2_ nanocomposites from solid state to melt state over a wide temperature range were investigated by mechanical spectroscopy. The purpose of the study is to reveal the mechanism of local and larger-scale segmental dynamics in PMMA/SiO_2_ nanocomposites with different compositions.

## 2. Materials and Methods

### 2.1. Materials’ Preparation

PMMA (*M_w_* = 1.20 × 10^5^ g/mol, *M_w_*/*M_n_* = 1.8) was obtained from Sigma-Aldrich (Shanghai, China). Silica nanoparticles (VK-SP15) with an average primary particle diameter of 15 nm and a BET surface area of 215 m^2^/g were purchased from Hangzhou Wanjing New Material Co., Ltd., Hangzhou, China. The polymer nanocomposites with various silica concentrations ($\phi_{{\mathrm{SiO}}_{2}}$ = 0.1, 0.5, 0.7, 1.0, 1.5, and 2 wt %) were prepared by a solution mixing method. The samples are denoted as PMMA/SiO_2_-0.1%, PMMA/SiO_2_-0.5%, and so on. In order to ensure the uniform dispersion of silica nanoparticles in polymer matrix, the particles were dispersed in *N*,*N*-dimethylacetamide (J&K Scientific LTD., Shanghai, China) and ultrasonicated for 30 min in a water bath. Then, PMMA was added to the solution in desired amounts and mixed by the magnetic stirrer. Finally, the samples were dried in a vacuum oven at 50 °C for two days to completely remove the solvent.

### 2.2. Characterization

The morphology and distribution of silica nanoparticles were characterized by transmission electron microscope (TEM, JEM2010, JEOL, Japan). The Fourier transition infrared spectra (FTIR) were measured to examine the interaction between PMMA and SiO_2_ nanoparticles using a Thermo-Nicolet (NEXUS G2192) spectrometer (Thermo Nicolet Corporation, Waltham, MA, US) at room temperature. For all samples, the spectra were taken in the wavenumber range from 400 to 4000 cm^−1^ with a resolution of 4 cm^−1^. The *T_g_* values of pure PMMA and PMMA/SiO_2_ nanocomposites were obtained by means of the differential scanning calorimeter (DSC, Q2000, TA Instruments, New Castle, DE, US) at a heating rate of 10 °C/min over 30 to 200 °C under nitrogen atmosphere.

To investigate the segmental relaxation dynamics of PMMA/SiO_2_ nanocomposites in the glass–rubber softening dispersion, the mechanical spectroscopy measurements were obtained by the forced-vibration method on a modified low-frequency inverted torsion pendulum. The detailed information of the instrument has been provided elsewhere [35]. The nonisothermal measurements of mechanical loss tangent (tan *δ*) and relative modulus (*G*) of the composites were measured at fixed frequencies of 0.05, 0.1, 0.5, 1.0, 2.0, and 4.0 Hz with a cooling rate of 1 K min^−1^ from 80 to 250 °C after preheating at 250 °C for two hours to ensure complete melting of samples. The isothermal measurements were carried out over a frequency range from 2 × 10^−1^ to 60 Hz at constant temperatures. All the mechanical measurements were performed under argon atmosphere with a pressure of 0.1 MPa to protect the samples from degradation and oxidation.

## 3. Results and Discussion

### 3.1. The Morphology and Interaction Characterization of PMMA/SiO_2_ Nanocomposites

**Figure 1:**
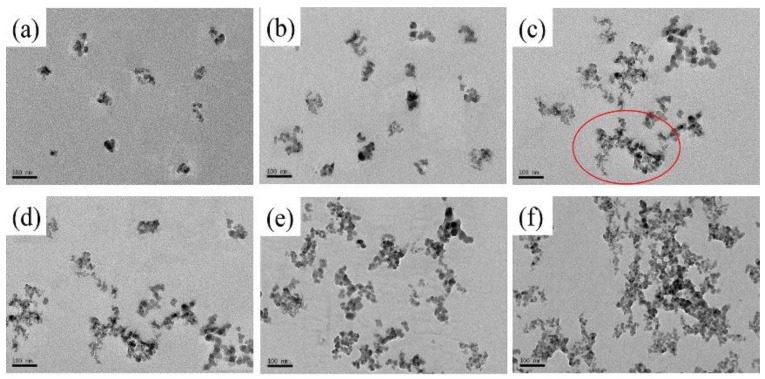
TEM images of poly(methyl methacrylate)/silica (PMMA/SiO_2_) polymer nanocomposites with different silica contents: (**a**) PMMA/SiO_2_-0.1%; (**b**) PMMA/SiO_2_-0.5%; (**c**) PMMA/SiO_2_-0.7%; (**d**) PMMA/SiO_2_-1.0%; (**e**) PMMA/SiO_2_-1.5%; (**f**) PMMA/SiO_2_-2.0%.

Figure 1 shows the TEM images of PMMA/SiO_2_ nanocomposites with different compositions. It can be seen that the silica nanoparticles are uniformly dispersed in the polymer matrix when $\phi_{{\mathrm{SiO}}_{2}}$ ≤ 0.5%, while the particles tend to agglomerate at $\phi_{{\mathrm{SiO}}_{2}}$ ≥ 0.7%. The aggregation also leads to the uneven distribution of silica nanoparticles.

**Figure 2:**
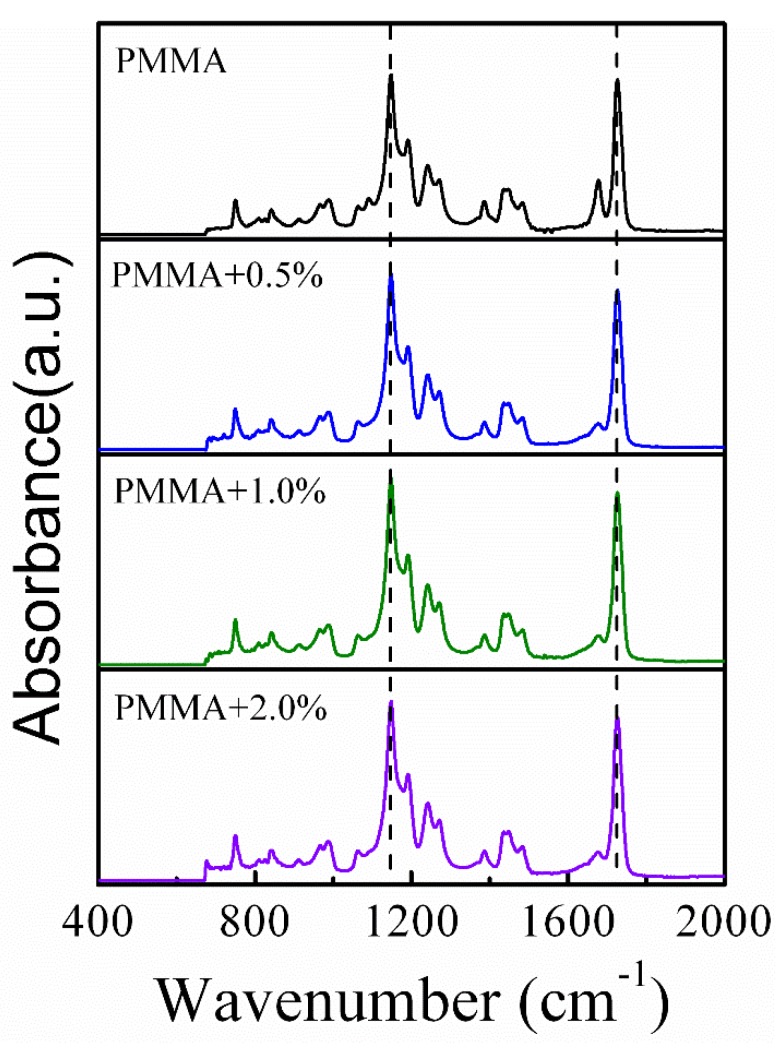
FTIR spectra of PMMA/SiO_2_ polymer nanocomposites with different silica contents.

It is expected that hydroxyl groups on the silica surface and the carbonyl groups of the polymer will form hydrogen bonding interactions between PMMA and silica nanoparticles. Figure 2 shows the FTIR spectra of PMMA/SiO_2_ nanocomposites with different silica contents. The absorption band located around 1736 cm^−1^ can be observed from the spectra, which is attributed to the vibrations of the carbonyl groups in PMMA. As reported in previous literature, the absorption band will broaden or shift in frequency if the carbonyl groups of polymer form hydrogen bonding with nanoparticles [30]. However, as Figure 2 shows, the position and width of the carbonyl groups’ absorption band shows no obvious change in PMMA/SiO_2_ nanocomposites, indicating a weak interaction between silica nanoparticles and PMMA. This may be because the silica nanoparticles are too small to spontaneously agglomerate due to their large surface energy. This is may be the reason why the silica nanoparticles begin to aggregate in the polymer matrix when $\phi_{{\mathrm{SiO}}_{2}}$ ≥ 0.7%.

### 3.2. The Glass Transition of PMMA/SiO_2_ Nanocomposites

**Figure 3:**
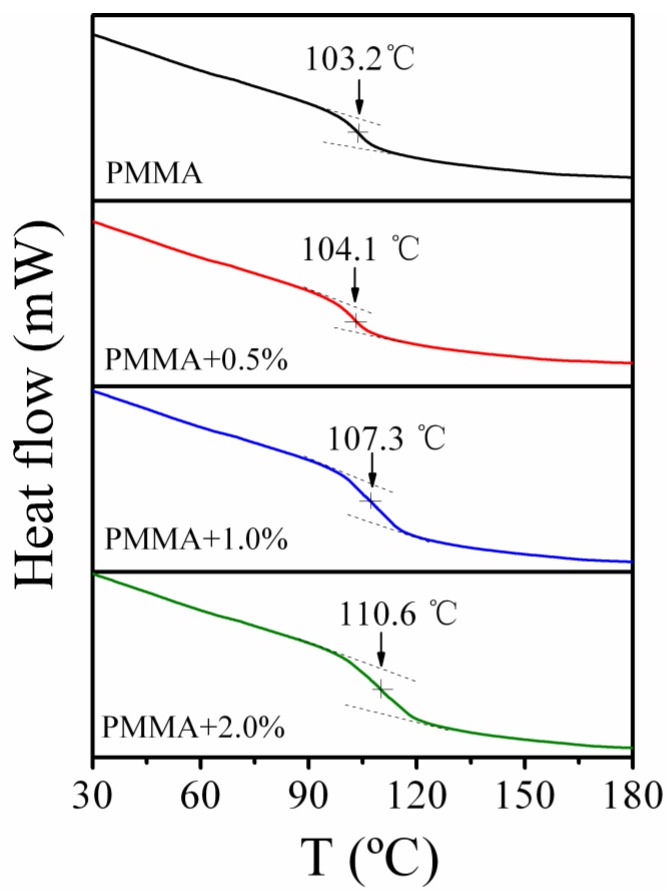
Differential scanning calorimeter (DSC) thermal analysis curves of PMMA and PMMA/SiO_2_ polymer nanocomposites with different silica contents.

Figure 3 shows the DSC results of PMMA/SiO_2_ nanocomposites with different compositions. For pure PMMA, the value of *T_g_* is about 103.2 °C. With increasing silica nanoparticles, the glass transition processes broaden and *T_g_* values shift to high temperatures, indicating the slowing down of relaxation dynamics. This is consistent with the previous works, which show that the increase of *T_g_* and slowing down of segmental dynamics can be observed even in the absence of interaction between the polymer and nanoparticles [36,37].

### 3.3. The Segmental Relaxation Dynamics of PMMA/SiO_2_ Nanocomposites

**Figure 4:**
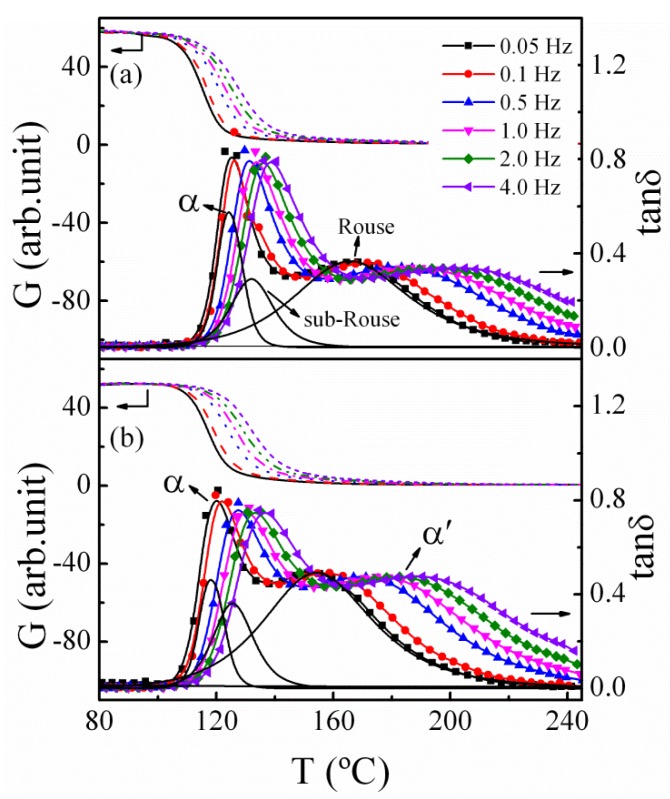
Temperature dependence of mechanical spectra (storage modulus, *G*, and mechanical loss, tan *δ*) for (**a**) PMMA and (**b**) PMMA/SiO_2_-0.7%. The solid lines are the fitting of the *α* and *α*’ peaks, corresponding to *α* relaxation, sub-Rouse modes, and Rouse modes.

For the sake of further study on the segmental relaxation dynamics of the polymer nanocomposites, we carried out mechanical spectroscopy measurements over a wide temperature range. Previous studies show that compared with the loss modulus *G”* and storage modulus *G*, the mechanical loss tangent is more sensitive to the relaxation modes involving longer-chain segmental motion [20,38]. Figure 4 shows the temperature dependence of mechanical loss (tan *δ*) and relative modulus (*G*) for pure PMMA and a typical PMMA/SiO_2_-0.7% nanocomposite at different frequencies from 80 to 250 °C. For both samples, the tan *δ* spectra exhibit an asymmetrical double-peak structure, labeled as the *α* and *α*’ peaks. From Figure 4, the *α* peak temperatures of pure PMMA and PMMA/SiO_2_-0.7% nanocomposite are roughly equal to their *T_g_* values determined by DSC, so the *α* relaxation process is related to the local segmental mode, which is ascribed to the cooperative rearrangement of chain segments involving local motion of only a few backbone bonds. On the other hand, the mechanical loss *α*’ peak at higher temperature, which is composed of the sub-Rouse modes and the Rouse modes, is associated with the motion of longer-chain segments [39], which usually involves the motion of 10 to 50 or more backbone bonds [40]. Because of the overlap and coupling of different molecular modes, it is difficult to resolve the contribution of each mode. Here, according to a nonlinear fitting method, the asymmetrical structure could be fitted well by three peaks (*α*, sub-Rouse, and Rouse modes) with distributions in relaxation time [39,41]. Details of the fitting results are given only at 0.05 Hz for clarity, as shown in Figure 4.

**Figure 5:**
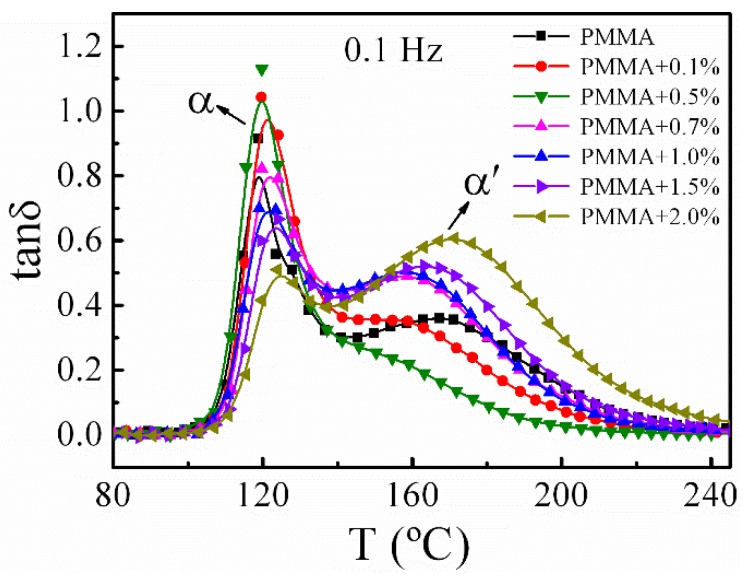
Temperature dependence of mechanical spectra for PMMA and PMMA/SiO_2_ polymer nanocomposites with different silica contents at 0.1 Hz.

**Figure 6:**
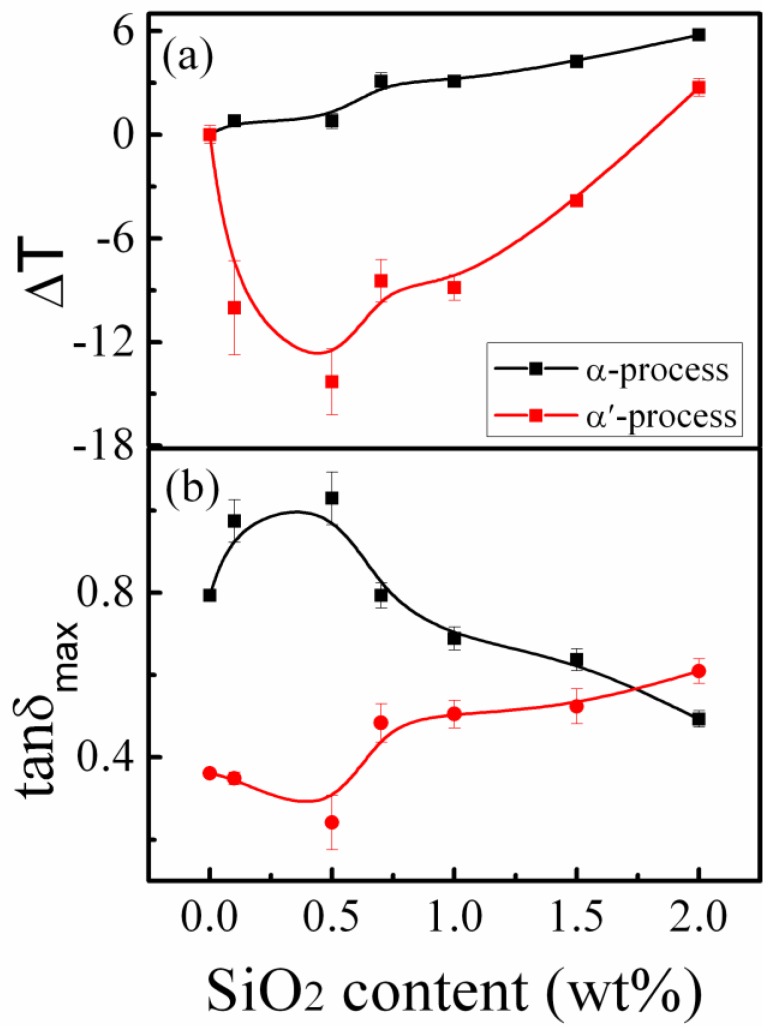
Variation of (**a**) the peak temperature deviations and (**b**) the peak heights as a function of silica nanoparticle content for *α* and *α*’ relaxations at the fixed frequency of 0.1 Hz. The solid lines were drawn as a guide to the eye.

Figure 5 shows the mechanical spectra for pure PMMA and PMMA/SiO_2_ nanocomposites with different components at 0.1 Hz. It can be seen that the polymer nanocomposites all exhibit the asymmetrical loss structure composed of *α* and *α*’ relaxation processes. Note that the peak temperature and height of these two peaks show strong composition dependence. The variation of the *α* and *α*’ relaxation peak temperature deviations (Δ*T*) and peak heights (tan *δ*_max_) of PMMA/SiO_2_ nanocomposites with different silica contents at 0.1 Hz are shown in Figure 6a,b, respectively. When $\phi_{{\mathrm{SiO}}_{2}}$ ≤ 0.5%, the *α* peak temperature hardly changes, and until $\phi_{{\mathrm{SiO}}_{2}}$ ≥ 0.7%, the *α* peak temperature increases with the increasing silica loading, and increases by about 6 °C at $\phi_{{\mathrm{SiO}}_{2}}$ = 2.0%. This result is agreement with the result obtained by DSC. However, the *α*’ relaxation peak shows a different behavior to the *α* process. The *α*’ peak temperature firstly decreases and drops by 15 °C at $\phi_{{\mathrm{SiO}}_{2}}$ = 0.5%, while at $\phi_{{\mathrm{SiO}}_{2}}$ ≥ 0.7%, it begins to increase. As for the peak height in Figure 6b, the value of the *α* process increases with the increase of silica content when $\phi_{{\mathrm{SiO}}_{2}}$ ≤ 0.5%, but when $\phi_{{\mathrm{SiO}}_{2}}$ ≥ 0.7%, tan *δ*_max_ decreases. On the contrary, the *α*’ peak height firstly decreases and then increases with the increase of silica content.

It is accepted that the relaxation peak temperature in the mechanical loss spectra partly reflects the rotational or reptational energy barrier of polymer segments, and the low transition temperature indicates a lower energy barrier [42]. The addition of silica nanoparticles to the PMMA matrix has two effects on the motion of chain segments, i.e., free volume and steric hindrance. As the *α* relaxation involves only a few backbone bonds and the weak interaction between the nanoparticles and polymer, the nanoparticles have little influence on the dynamics of *α* relaxation at low silica loading and there is no obvious change in the peak temperature. Lin et al. [33] also reported that introduction of silica nanoparticles has no significant effect on *α* relaxation dynamics due to the absence of interaction between silica and polymer, which is consistent with the results presented here. However, the *α*’ relaxation process associated with the longer-chain segmental relaxation shows strong composition dependence. At $\phi_{{\mathrm{SiO}}_{2}}$ ≤ 0.5%, the nanoparticles are uniformly dispersed in the PMMA matrix, as shown by TEM images, and effectively increase the distance between adjacent PMMA chains. Therefore, the polymer chains easily relax due to the increase of free volume, leading to the decrease of *α*’ relaxation temperature. However, the higher content of silica nanoparticles leads to agglomeration and undoubtedly induces steric obstacles to hinder the segmental motion, resulting in the increase of the required energy barrier.

**Figure 7:**
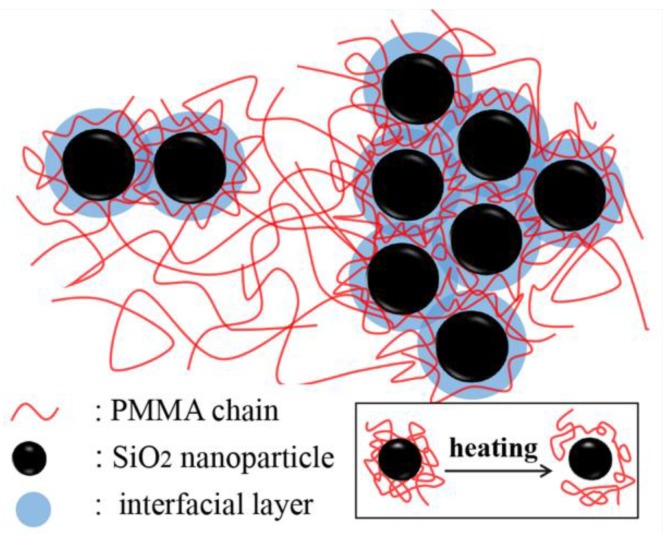
Schematic illustration of heterogeneous distribution of polymer chains in PMMA/SiO_2_ polymer nanocomposites at high silica loading. The inset shows the evolution of polymer chains’ state around nanoparticles upon heating.

On the other hand, the height of relaxation peak generally reflects the concentration of motion units involved in the relaxation. Figure 6b suggests that the concentration of motion units of *α* relaxation first increased and then decreased with increasing silica nanoparticle content, while the *α*’ relaxation process shows the opposite behavior. At low silica nanoparticle loading, the motion units of *α* relaxation contain only a few backbone bonds (about 1–2 nm long), which can easily bypass the nanoparticles, and more local segments of PMMA can relax due to the larger free volume induced by the nanoparticles. However, the *α*’ relaxation associated with the longer-chain segmental relaxation involves 10–50 or more backbone bonds (about several tens of nanometers in length), which makes it more difficult to bypass the nanoparticles, leading to the decrease of *α*’ relaxation motion units. However, as Holt et al. and Fragiadakis et al. [14,43] reported, the interfacial layer (about 2–4 nm) could be formed at high nanoparticle loading, owing to the physical adsorption effect, even in the absence of attraction between the polymer and nanoparticles. The length-scale of motion units involved in *α* relaxation is about 1–2 nm, so the *α* relaxation units were bonded to nanoparticles, resulting in the decrease of the concentration of motion units involved in *α* relaxation at high silica loading. However, as temperature increases, the bonds between polymer chains and nanoparticles gradually dissociate, as shown in the inset of Figure 7. Further, at high silica content, the inhomogeneous distribution of silica nanoparticles in PMMA leads to the strong overlap of interfacial layer regions and the local heterogeneity of polymer chain concentration, as illustrated in Figure 7. With increasing temperature, the longer-chain clusters at the overlapped interface layers with larger polymer chain density than the matrix begin to relax and dissociate. This may be the reason why the concentration of relaxation units of the *α*’ process increases at $\phi_{{\mathrm{SiO}}_{2}}$ ≥ 0.7%. This is consistent with the result of Lin et al. [33], who found that the segmental relaxation peak intensity of the interfacial layer increases with increasing temperature.

**Figure 8:**
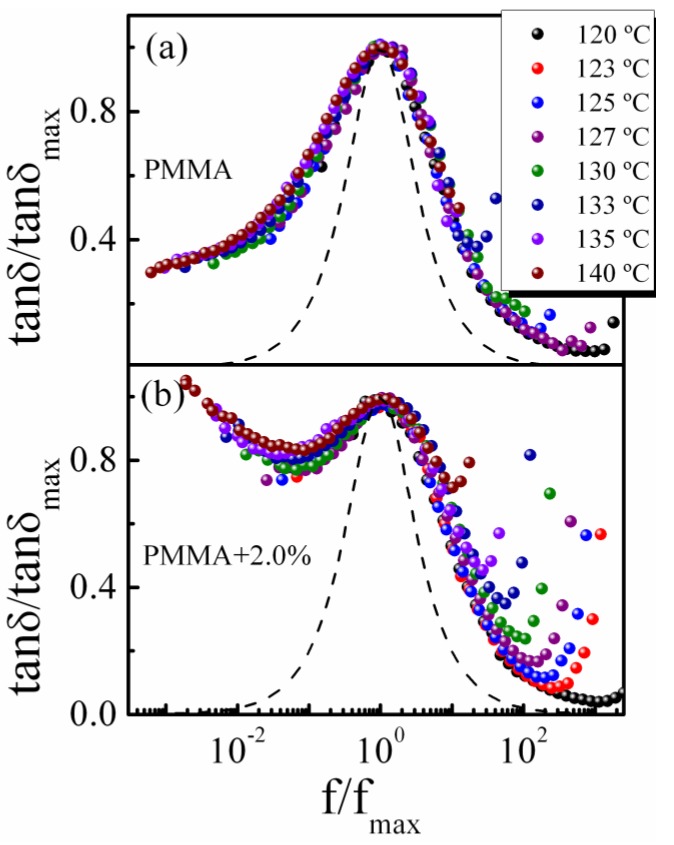
The normalized master curves of (**a**) PMMA and (**b**) PMMA/SiO_2_-2% at different temperatures.

**Figure 9:**
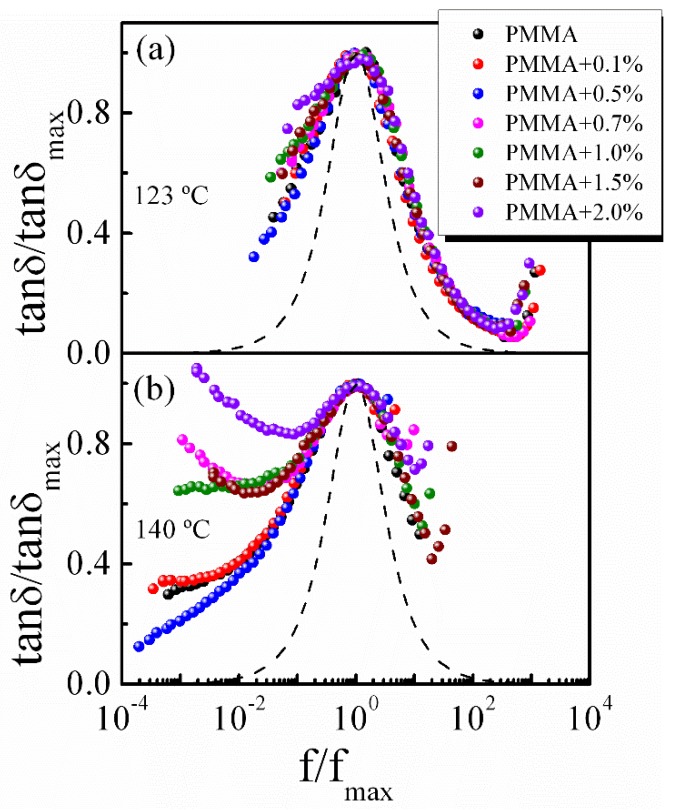
The normalized master curves of PMMA/SiO_2_ polymer nanocomposites with different silica contents at (**a**) 123 °C and (**b**) 140 °C.

The time–temperature superposition (TTS) is the fundamental principle usually used for analyzing the dynamics of supercooled liquid and polymers. The validity of TTS indicates that the change of temperature moves only the time scale of the entire mechanical response and does not affect the shape of the loss spectrum [44,45]. The normalized master curves of the loss tangent tan *δ*/tan *δ*_max_ as a function of *f*/*f*_max_ for pure PMMA and PMMA-2% SiO_2_ are shown in Figure 8. As observed, the curves fail to superpose at high- and low-frequency sides, indicating that the TTS principle is invalid over the entire temperature range, which results from the different temperature dependence of various relaxation modes in the polymer [20,46]. Note that the normalized master curves of relaxation peaks are broader than the classical Debye response, indicating a broader relaxation time distribution. The normalized master curves of PMMA/SiO_2_ nanocomposites with different compositions are compared at 123 and 140 °C in Figure 9. Compared with the relaxation peaks of the system at 123 °C, the width broadening at 140 °C is more pronounced as the content of silica increases. Further, the mechanical loss spectra at the low-frequency side, which is related to the *α*’ relaxation, deviates obviously from a standard Debye behavior, especially for composites with $\phi_{{\mathrm{SiO}}_{2}}$ ≥ 0.7%. This feature demonstrates that the addition of silica nanoparticles broadens the relaxation time distribution and influences the movement environment of relaxing species in the polymer nanocomposites, particularly for *α*’ relaxation and a high filler system. According to the theoretical approaches proposed by Nakazawa et al. [47,48], the width broadening of the mechanical spectra may result from the local heterogeneity, which is consistent with the above analysis.

**Figure 10:**
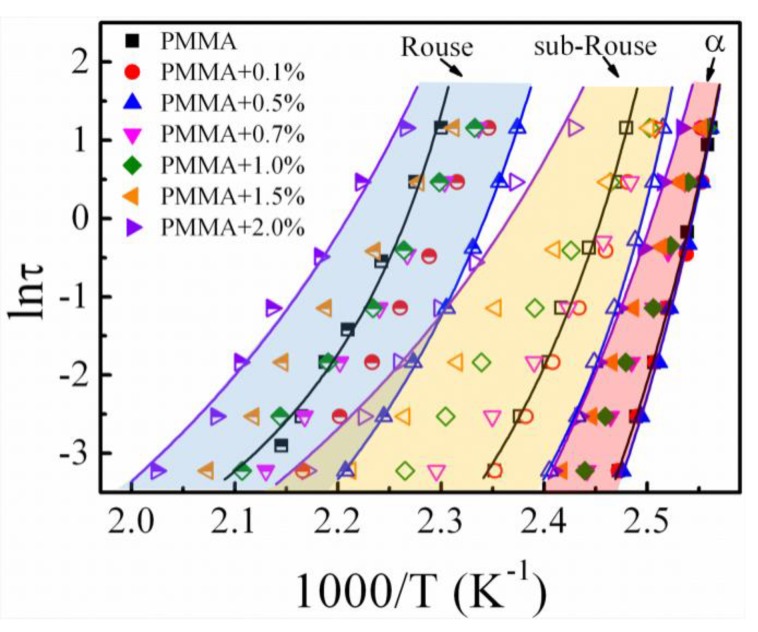
Temperature dependence of relaxation times (*τ*) of different modes for PMMA/SiO_2_ polymer nanocomposites with different silica contents.

To quantitatively study the impact of silica nanoparticle loading on the segmental relaxation, the relaxation times, *τ*, of the *α* relaxation process and sub-Rouse and Rouse relaxation modes for pure PMMA and PMMA/SiO_2_ polymer nanocomposites were calculated from the corresponding frequency and temperature at those relaxation peaks in the temperature-dependent mechanical spectra at different frequencies, as shown in Figure 4, and the temperature dependence of relaxation times is shown in Figure 10. The relation deviates strongly from the simple thermally activated behavior or Arrhenius behavior. Generally, it could be well fitted by the Vogel–Fulcher–Tamman (VFT) equation [49,50,51]:

(1) $$\tau =\tau_{0}\exp\left[\frac{1}{\alpha_{f}\left(T-T_{0}\right)}\right]$$

where *τ*_0_ is the pre-exponential factor, *α_f_* is the thermal expansion coefficient of the fractional free volume, and *T*_0_ is the critical temperature (or “Vogel temperature”) at which *τ* diverges. To reduce the uncertainty of VFT equation fitting in the limited frequency range, according to previous works, the values of ln*τ_0_* of the *α* process, sub-Rouse modes, and Rouse modes are assumed about to be −14, −10, and −6.9 s, respectively [52,53]. As shown in Figure 10, the temperature dependences of relaxation time *τ* for different relaxation modes are well-described by a single VFT equation. For clarity, the details of the fitting results are given only for pure PMMA and PMMA/SiO_2_-0.5% and 2%. Extrapolation of *α* relaxation VFT fit to *τ* = 100 s estimates the *T_g_* (denoted as *T_g_*_,100s_). Furthermore, the dynamic fragility *m* could be determined from *α* relaxation VFT parameters as:

(2) $$m=\frac{{\left(\alpha_{f}T_{g}\right)}^{-1}}{\log{\left(1-T_{0}/T_{g}\right)}^{2}}$$

The obtained fitting parameters and fragility *m* are reported as a function of silica nanoparticle content in Table 1.

Fragility is known to be concerned with the interaction between the nanoparticles and polymer: its increase or decrease indicates the attractive or repulsive interaction, respectively [54]. According to the result shown in Table 1, *m* hardly changes at $\phi_{{\mathrm{SiO}}_{2}}$ ≤ 0.5%, but it increases significantly when $\phi_{{\mathrm{SiO}}_{2}}$ ≥ 0.7%, indicating the attractive interaction at high loading. This is consistent with previous results showing that the interfacial layer can be formed by a physical adsorption effect between the polymer and nanoparticles at high silica loading. The value of $\alpha_{f}$ is related to the sensitivity of the samples to temperature change [42]. We can find that $\alpha_{f,Rouse}$ > $\alpha_{f,sub-Rouse}$ > $\alpha_{f,\alpha }$, indicating that the longer-chain segmental relaxation is more sensitive to the change of temperature. Moreover, it was also found that the temperature variation of longer-chain segmental relaxation time is larger than that of local segmental relaxation with increasing silica content, proving that the longer-chain segmental motion is more sensitive to the addition of silica nanoparticles. This result is consistent with a previous study which reported that the *α* relaxation is more significantly affected by the filler in comparison to *β* relaxation (related with the side-chain rational motion) due to the smaller side chains experiencing little obstruction [55].

For all relaxation processes, $\alpha_{f}$ increases with the increase of the silica content and decreases when $\phi_{{\mathrm{SiO}}_{2}}$ ≥ 0.7%. Based on the free volume theory, the fractional free volume $V_{f}$ of polymers above *T_g_* can be expressed approximately as follows [56]:

(3) $$V_{f}=V_{g}+\alpha_{f,\alpha }\left(T-T_{g}\right),$$

where $V_{g}$ is the fractional free volume of polymer at *T_g_*. According to the relation, we obtained that the fractional free volume of longer-chain segmental relaxation is larger than the *α* relaxation process, due to the bigger value of $\alpha_{f}$. The variation of this fractional free volume is consistent with the result of positron annihilation lifetime spectroscopy, which reported that the fractional free volume increases rapidly above *T_ll_* (*T_α’_*) for cured chlorinated butyl rubber [56]. The variation of $\alpha_{f}$ suggests that a small amount of silica nanoparticles can increase the free volume, leading to the increase of segmental mobility. However, the free volume of the polymer nanocomposites decreases when $\phi_{{\mathrm{SiO}}_{2}}$ ≥ 0.7%, which is agreement with the results obtained by nonisothermal mechanical spectra.

## 4. Conclusions

The local and larger-scale segmental relaxation dynamics of PMMA/SiO_2_ nanocomposites with different silica contents over a wide temperature range were investigated by low-frequency mechanical spectroscopy combined with TEM, FTIR, and DSC. For the local *α* relaxation, with the increasing silica content, the peak temperature hardly changes at $\phi_{{\mathrm{SiO}}_{2}}$ ≤ 0.5% and increases at $\phi_{{\mathrm{SiO}}_{2}}$ ≥ 0.7%, while the peak height firstly increases at $\phi_{{\mathrm{SiO}}_{2}}$ ≤ 0.5% and then decreases. For the larger-scale *α*’ relaxation, composed of sub-Rouse modes and Rouse modes, both the peak temperature and peak height firstly decrease at $\phi_{{\mathrm{SiO}}_{2}}$ ≤ 0.5% and then increase at $\phi_{{\mathrm{SiO}}_{2}}$ ≥ 0.7%. At $\phi_{{\mathrm{SiO}}_{2}}$ ≤ 0.5%, the nanoparticles are uniformly dispersed in the PMMA matrix, and the induced free volume leads to the decrease of peak temperature of *α*’ relaxation. Meanwhile, the short segments of PMMA related to *α* relaxation can easily bypass the particles, but the longer segments related to *α*’ relaxation cannot, resulting in the opposite behaviors of relaxation unit concentration for the peak heights. However, at $\phi_{{\mathrm{SiO}}_{2}}$ ≥ 0.7%, the polymer segments were bonded to the nanoparticles due to the physical adsorption effect, leading to the decrease of relaxation unit concentration involved in *α* relaxation. However, the dissociation of those bonds with heating and the local heterogeneity of polymer chain concentration due to the overlapped interfacial layer can result in the abnormal increase of relaxation unit concentration for *α*’ relaxation. In addition, the time–temperature superposition is invalid for the polymer nanocomposites over the entire temperature range due to the different temperature dependence of relaxation modes, and the larger-scale relaxation shows a larger fractional free volume than *α* relaxation.

## Figures and Tables

**Table 1:** The fitting parameters of different relaxation modes of PMMA/SiO_2_ polymer nanocomposites.

Silica Content	*α* Process		Sub-Rouse			Rouse	
	$\alpha_{f\;}\;(K^{-1})$	T_0_ (K)	*m*	$\alpha_{f}\;(K^{-1})$	T_0_ (K)	$\alpha_{f\;}\;(K^{-1})$	T_0_ (K)
0 wt %	1.41 × 10^−3^	349	230	2.50 × 10^−3^	367	3.65 × 10^−3^	402
0.1 wt %	1.47 × 10^−3^	351	228	2.55 × 10^−3^	358	3.66 × 10^−3^	393
0.5 wt %	1.49 × 10^−3^	351	229	3.12 × 10^−3^	369	4.26 × 10^−3^	392
0.7 wt %	1.13 × 10^−3^	353	321	1.62 × 10^−3^	344	3.10 × 10^−3^	389
1.0 wt %	1.04 × 10^−3^	354	336	1.31 × 10^−3^	332	2.85 × 10^−3^	386
1.5 wt %	9.79 × 10^−4^	357	369	1.15 × 10^−3^	316	2.50 × 10^−3^	383
2.0 wt %	9.22 × 10^−4^	358	421	1.06 × 10^−3^	336	2.42 × 10^−3^	392
